# Supplementary material for: Characteristic of K3 (CpG-ODN) as a Transcutaneous Vaccine Formulation Adjuvant
Source: Pharmaceutics. 2020 Mar 15;12(3):267. doi: 10.3390/pharmaceutics12030267 (PMC7151019; doi:10.3390/pharmaceutics12030267)
Supplement: Supplementary file 1 [file pharmaceutics-12-00267-s001.pdf]

pharmaceutics

MDPI

Supplementary Materials: Characteristic of K3 (CpG-ODN) as a Transcutaneous Vaccine Formulation Adjuvant

Antibody Reagents Used in Flow Cytometry Analysis

The skin cells were stained with the following antibodies: APC anti-MHC class II (clone AF6-120.1; BioLegend, San Diego, CA, USA), APC anti-CD11c (clone N418, BD), PE anti-TLR1 (clone eBio TR23), PE anti-TLR2 (clone 6C2), PE anti-TLR3 (clone 40C1285.6), PE anti-TLR4 (clone 76B357.1), PE anti-TLR5 (clone 85B152.5), PE anti-TLR6 (clone 418601), PE anti-TLR7, PE anti-TLR8 (clone 44C143), and PE anti-TLR9 (clone 26C593.2) from Novus Biologicals (Centennial, CO, USA). The cell suspensions were analyzed by flow cytometry after adding 7-ADD (BioLegend) to stain dead cells. Differentiation and activation of T- and B-cells were stained with the following antibodies: FITC anti-IgG1 (polyclone) from Southern Biotech (Birmingham, AL). PE anti-IgD (clone 11-26c. 2a) and eFluor 450 anti-CD4 (clone GK1.5) from Thermo Fisher Scientific. PE anti-CD80 (clone 16-10A1), PE-Cy7 anti-CD95 (clone Jo2), APC anti-CD138 (clone 281-2) and APC-Cy7 anti-B220 (clone RA3-6B2) from BD. PE-Cy7 anti-CD62L (clone MEL-14), APC anti-CD127 (clone A7R34), APC anti-CD86 (clone GL-1), APC-Cy7 anti-CD44 (clone IM7) and Alexa Fluor 647 anti-GL7 (clone GL7) and BV421 anti-B220 (clone RA3-6B2) from BioLegend. VioBlue anti-CD38 (clone 90.4) from Miltenyi Biotec. The dead cells were stained using Zombie Green and Zombie Aqua from BioLegend for 15 min.

The OVA-specific $CD8^+$ T cells were detected with the following antibodies: FITC anti-CD8 (clone KT15), PE-Cy5 anti-CD19 (clone 6D5) and APC Pro5 MHC Class I pentamers from ProImmune Limited (United Kingdom). The dead cells were stained using Zombie Aqua from BioLegend for 15 min. These cells were stained according to the manufacturers' protocols.

The T cells used for the proliferation assay were stained with the following antibodies: FITC anti-CD45.1 (clone A20) from BioLegend; eFluor 450 anti-CD4 (clone GK1.5) and PE-Cy7 anti-CD8α (clone 53-6.7) from Thermo Fisher Scientific; and V450 anti-Thy1.1 (clone OX-7) from BD. The dead cells were stained using Zombie Aqua from BioLegend for 15 min.

Immunofluorescence Staining of Germinal Center

Mice were immunized with OVA and K3 using the poke-and-patch method twice at 2-week intervals, and DLNs were collected 2 weeks after the second immunization for observing the germinal center. These tissue samples were immersed in 4% PFA/PBS overnight at 4°C. After a sucrose hydration treatment, the skin and DLNs were frozen in OCT compound (Sakura Finetek) and cut into 8-μm-thick sections using a cryostat (Leica Biosystems). After the OCT compound was removed, samples were blocked with 5% goat serum/PBS at room temperature for 1 h. The germinal center was stained by the direct method using the following antibodies. Various antibody solutions were prepared with antibody dilution buffer (1% BSA, 0.3% Triton, 1×PBS), added dropwise to the samples and incubated overnight at 4°C. The germinal center was stained with the following antibodies: Fluorescein anti-PNA from Vector Laboratories (Burlingame, CA), Alexa Fluor 594 anti-CD3 (clone 17A2), and Alexa Fluor 647 anti-IgD (clone 11-26c.2a) from BioLegend.

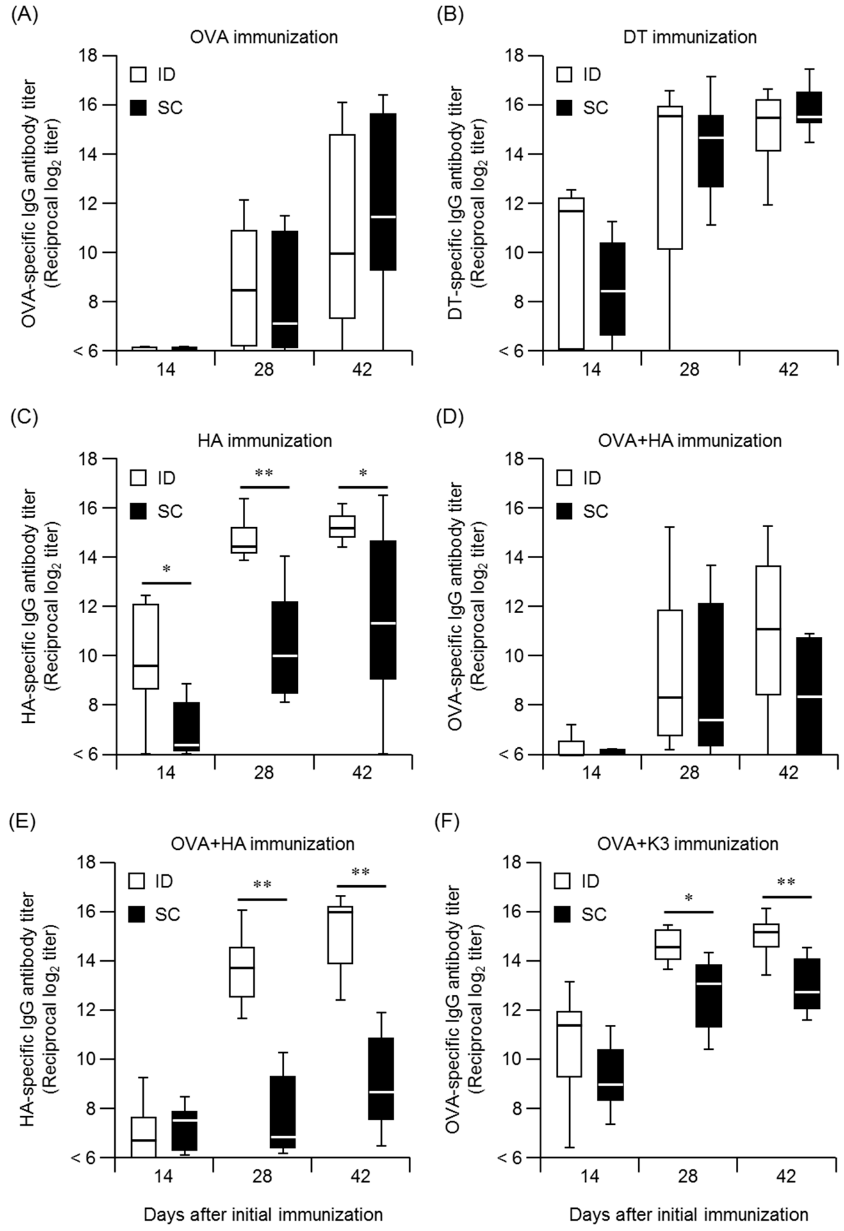

Figure S1. Difference in effect of antigen-specific immune responses administrated by subcutaneous or intradermal immunization.

To investigate the immune response from various antigens, mice were intradermally or subcutaneously immunized with 10 µg OVA (A), 1 µg diphtheria toxin (DT) (B), 10 µg influenza hemagglutinin (HA) antigen derived from A/California/7/2009 (C), 10 µg OVA plus 10 µg HA (D, E) or 10 µg OVA plus 20 µg K3 (F) solution at 50 µL/head, respectively. The immunization was conducted three times at 2-week intervals. Sera were collected after immunization and assayed for the antigen-specific IgG titer by ELISA. No change was observed in antibody production when OVA or DT antigen was administered by subcutaneous immunization (SC) or intradermal immunization (ID). ID of HA antigen showed higher antibody production than SC, suggesting that virus-derived proteins and nucleic acids contained in the HA antigen may function as adjuvants. When the OVA solution was mixed with the HA antigen or K3 and used for immunization, antibody production was enhanced by ID. In a solution in which HA antigen or K3 was mixed with OVA, antibody production was enhanced by ID when compared with SC. These results show that a combination of an adjuvant is necessary to induce an enhanced immune response when administering an antigen in the skin. The boxplot shows the median value with the 25th–75th percentiles and the error bars indicate the 5th–95th percentiles. Data were obtained from three mice. (Student's *t*-Test, $*p < 0.05$, $** \ p < 0.01$)

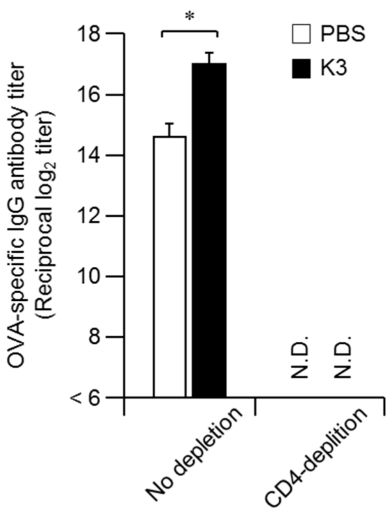

Figure S2. The contribution of CD4⁺ cells on induction of OVA-specific antibody production.

To evaluate the contribution of $CD4^+$ T cells in antigen-specific antibody production, C57BL/6 mice were injected with either 200 µg rat IgG antibody or 100 µg anti CD4 monoclonal antibody on day 1, 2, 3 and every subsequent 4 days. Depletion of $CD4^+$ T cells in the blood was confirmed by flow cytometry. These mice were transcutaneously immunized with 1 mg OVA-loaded HG with K3 or PBS on days 0, 14, and 28 using the poke-and-patch method, and then sera collected on day 42 were assayed to determine the OVA-specific IgG titer by ELISA. In the non-depleted group, the combined use of K3 induced a significantly higher antibody titer than the PBS group, and K3 adjuvant activity was observed. However, antigen-specific antibody production was completely lost by $CD4^+$ T cell depletion, indicating that the $CD4^+$ T cell helper function contributes to B cell differentiation in plasma cells and the transcutaneous immunization induction of antibody production. Data are expressed as mean ± SEM from results from three mice. (Student's *t*-Test, $*p < 0.05$)

Table S1. Blood and biochemical tests.

ALP (U/L)		Day				
		0	1	21	22	42
Placebo-sdMN	ID-1	242	226	196	211	199
	ID-2	148	174	154	156	152
K3-loaded sdMN	ID-3	175	204	152	171	162
	ID-4	296	321	286	297	290
	ID-5	299	300	264	290	287
	ID-6	387	433	343	436	366

ALT (U/L)		Day				
		0	1	21	22	42
Placebo-sdMN	ID-1	42	39	48	43	42
	ID-2	13	14	13	9	9
K3-loaded sdMN	ID-3	24	24	18	18	25
	ID-4	18	18	22	26	26
	ID-5	18	18	18	16	17
	ID-6	27	27	29	33	21

AST (U/L)		Day				
		0	1	21	22	42
Placebo-sdMN	ID-1	34	31	37	30	33
	ID-2	18	18	16	16	16
K3-loaded sdMN	ID-3	25	25	19	20	23
	ID-4	19	19	21	24	22
	ID-5	19	17	16	16	16
	ID-6	20	21	21	23	19

B-AMY (U/L)		Day				
		0	1	21	22	42
Placebo-sdMN	ID-1	83	79	80	81	82
	ID-2	51	48	56	45	40
K3-loaded sdMN	ID-3	93	88	81	95	97
	ID-4	100	99	89	90	96
	ID-5	87	82	83	76	70
	ID-6	52	50	52	56	54

BUN (mg/dL)		Day				
		0	1	21	22	42
Placebo-sdMN	ID-1	10	10	12	13	10
	ID-2	9	9	9	9	9
K3-loaded sdMN	ID-3	16	18	18	14	13
	ID-4	13	14	12	15	16
	ID-5	19	17	17	17	16
	ID-6	10	9	12	13	10

CPK (U/L)		Day				
		0	1	21	22	42
Placebo-sdMN	ID-1	203	195	61	62	62
	ID-2	116	102	87	82	87
K3-loaded sdMN	ID-3	142	147	118	124	113
	ID-4	128	128	120	132	135
	ID-5	167	171	154	150	138
	ID-6	136	129	157	155	194

CRE (mg/dL)		Day				
		0	1	21	22	42
Placebo-sdMN	ID-1	0.96	0.93	0.74	0.77	0.73
	ID-2	0.65	0.70	0.68	0.66	0.64
K3-loaded sdMN	ID-3	0.85	0.91	0.87	0.88	0.81
	ID-4	0.73	0.78	0.70	0.69	0.77
	ID-5	0.07	0.05	0.02	0.02	0.03
	ID-6	0.75	0.75	0.74	0.71	0.68

CRP (mg/dL)		Day				
		0	1	21	22	42
Placebo-sdMN	ID-1	0.10	0.08	0.02	0.02	0.04
	ID-2	0.02	0.01	0.01	0.01	0.09
K3-loaded sdMN	ID-3	0.03	0.03	0.01	0.01	0.02
	ID-4	0.03	0.02	0.74	0.46	0.01
	ID-5	0.07	0.05	0.02	0.02	0.03
	ID-6	0.08	0.49	0.02	0.02	0.04

GLU (mg/dL)		Day				
		0	1	21	22	42
Placebo-sdMN	ID-1	84	89	101	90	121
	ID-2	104	105	114	93	98
K3-loaded sdMN	ID-3	91	91	84	96	87
	ID-4	91	93	87	94	97
	ID-5	102	95	99	91	100
	ID-6	163	129	100	104	118

Hb (g/dL)		Day				
		0	1	21	22	42
Placebo-sdMN	ID-1	16	16	16	15	16
	ID-2	16	16	16	15	15
K3-loaded sdMN	ID-3	15	15	15	15	15
	ID-4	14	14	14	13	14
	ID-5	15	15	15	15	15
	ID-6	14	13	13	13	13

Hct (%)		Day				
		0	1	21	22	42
**Placebo-sdMN**	ID-1	50	49	49	48	48
	ID-2	49	48	49	46	47
**K3-loaded sdMN**	ID-3	47	46	46	46	44
	ID-4	45	44	41	41	44
	ID-5	44	44	43	43	44
	ID-6	41	39	39	39	36

LDH (U/L)		Day				
		0	1	21	22	42
**Placebo-sdMN**	ID-1	162	146	153	139	156
	ID-2	155	140	147	129	125
**K3-loaded sdMN**	ID-3	281	256	225	217	226
	ID-4	154	155	155	150	152
	ID-5	173	176	168	167	177
	ID-6	180	169	181	164	173

MCH (pg)		Day				
		0	1	21	22	42
**Placebo-sdMN**	ID-1	27	27	27	27	27
	ID-2	29	29	29	29	29
**K3-loaded sdMN**	ID-3	29	29	30	30	30
	ID-4	30	30	31	30	31
	ID-5	30	30	31	30	31
	ID-6	31	31	31	31	31

MCHC (g/dL)		Day				
		0	1	21	22	42
**Placebo-sdMN**	ID-1	32	32	33	32	32
	ID-2	32	32	33	32	32
**K3-loaded sdMN**	ID-3	33	32	33	33	34
	ID-4	32	33	33	32	33
	ID-5	34	34	35	34	35
	ID-6	34	34	34	35	35

MCV (fL)		Day				
		0	1	21	22	42
**Placebo-sdMN**	ID-1	83	84	83	84	83
	ID-2	90	89	89	89	89
**K3-loaded sdMN**	ID-3	89	90	90	90	88
	ID-4	94	92	93	93	93
	ID-5	89	89	88	88	88
	ID-6	89	90	89	89	89

MPV (fL)		Day				
		0	1	21	22	42
**Placebo-sdMN**	ID-1	11	10	10	10	10
	ID-2	10	10	10	10	10
**K3-loaded sdMN**	ID-3	11	10	10	10	10
	ID-4	8	8	8	8	8
	ID-5	10	10	10	10	10
	ID-6	9	9	9	9	9

PCT (ng/mL)		Day				
		0	1	21	22	42
**Placebo-sdMN**	ID-1	0.32	0.27	0.32	0.29	0.30
	ID-2	0.13	0.14	0.13	0.13	0.13
**K3-loaded sdMN**	ID-3	0.29	0.28	0.30	0.28	0.30
	ID-4	0.33	0.33	0.30	0.28	0.35
	ID-5	0.31	0.29	0.26	0.26	0.30
	ID-6	0.34	0.33	0.38	0.37	0.31

PDW (%)		Day				
		0	1	21	22	42
**Placebo-sdMN**	ID-1	13	12	13	13	11
	ID-2	13	11	12	11	12
**K3-loaded sdMN**	ID-3	12	12	12	11	11
	ID-4	9	8	8	8	9
	ID-5	12	11	11	12	12
	ID-6	9	9	9	9	9

PLT ($\times 10^4/\mu L$)		Day				
		0	1	21	22	42
**Placebo-sdMN**	ID-1	30	27	30	28	31
	ID-2	13	13	13	12	13
**K3-loaded sdMN**	ID-3	27	28	30	29	30
	ID-4	40	40	36	35	41
	ID-5	31	30	26	25	29
	ID-6	40	38	43	42	35

RBC ($\times 10^4/\mu L$)		Day				
		0	1	21	22	42
**Placebo-sdMN**	ID-1	599	577	589	572	584
	ID-2	541	545	548	518	526
**K3-loaded sdMN**	ID-3	529	510	516	516	498
	ID-4	477	472	446	438	469
	ID-5	494	492	491	484	502
	ID-6	454	432	434	433	408

RDW (%)		Day				
		0	1	21	22	42
**Placebo-sdMN**	ID-1	19	19	19	19	19
	ID-2	13	12	12	12	12
**K3-loaded sdMN**	ID-3	12	12	12	12	12
	ID-4	12	12	12	12	12
	ID-5	12	12	12	12	12
	ID-6	12	12	12	12	12

T-Bil (mg/dL)		Day				
		0	1	21	22	42
**Placebo-sdMN**	ID-1	0.89	0.72	0.56	0.74	0.86
	ID-2	0.99	1.30	0.97	1.16	1.10
**K3-loaded sdMN**	ID-3	0.74	0.82	0.75	0.66	0.83
	ID-4	0.55	0.68	0.53	0.63	0.58
	ID-5	0.76	1.43	1.03	1.51	1.18
	ID-6	0.60	0.50	0.70	0.52	0.61

WBC ($/\mu L$)		Day				
		0	1	21	22	42
**Placebo-sdMN**	ID-1	9360	8920	8410	9640	9750
	ID-2	3260	3140	3760	3260	2950
**K3-loaded sdMN**	ID-3	6310	6550	5690	6560	6990
	ID-4	5980	6510	7440	5540	6520
	ID-5	5920	7390	5800	6810	6930
	ID-6	7020	7370	4490	5450	4900
